# Diversity of salt tolerance in *Vigna riukiuensis*

**DOI:** 10.1270/jsbbs.25064

**Published:** 2026-06-11

**Authors:** Fanmiao Wang, Yurie Iki, Keitaro Tanoi, Ken Naito

**Affiliations:** 1 Research Center of Genetic Resources, National Agriculture and Food Research Organization, 2-1-2 Kannondai, Tsukuba, Ibaraki 305-8602, Japan; 2 Graduate School of Frontier Sciences, The University of Tokyo, 5-1-5 Kashiwanoha, Kashiwa City, Chiba 277-8561, Japan; 3 Graduate School of Agricultural and Life Sciences, The University of Tokyo, 1-1-1 Yayoi, Bunkyo, Tokyo 113-8657, Japan

**Keywords:** *Vigna riukiuensis*, crop wild relative, salt tolerance, chlorophyll fluorescence

## Abstract

*Vigna riukiuensis* is one of the most salt-tolerant species within the genus *Vigna*, yet intraspecific variation in its salt tolerance remains uncharacterized. In this study, we evaluated 61 accessions of *V. riukiuensis* under 200 mM NaCl using effective quantum yield (Y(II)) as an indicator of salt tolerance. Accessions were classified into five clusters, showing substantial variation that accessions in the tolerant group maintained Y(II) for four weeks whereas those in the sensitive group lost Y(II) in a week. We repeated the Y(II)-based classification for a subset of accessions and the results generally reproduced that of the first evaluation. In addition, we measured Na^+^ and K^+^ allocation in roots, stems and leaves and found the pattern of Na^+^ allocation greatly varied even within the most tolerant accessions whereas the pattern of K^+^ allocation, not the Na^+^/K^+^ ratio, showed a minor correlation with salt tolerance. Interestingly, JP254554 restricted Na^+^ transport from roots to leaves, but it did not develop Casparian strips even under salt stress. This study provides the first comprehensive assessment of intraspecific variation in *V. riukiuensis*, identifying promising donor accessions for breeding, and highlighting various unknown mechanisms to be elucidated in the future.

## Introduction

*Vigna riukiuensis* is one of the most salt-tolerant species in the genus *Vigna*. It shows higher relative quantum yield and shoot biomass than adzuki bean (*Vigna angularisis*), an important legume crop in East Asia (Iseki *et al.* 2016). As *V. riukiuensis* is a wild relative of and cross-compatible with adzuki bean (Ogiso-Tanaka *et al.* 2023), it can be a donor parent for improving salt tolerance of adzuki bean. For this purpose, it is necessary to evaluate salt tolerance and select suitable accessions, as well as to understand the mechanisms of salt tolerance in *V. riukiuensis*.

In fact, Noda *et al.* (2022, 2023) has revealed one of the accessions of *V. riukiuensis*, ‘Tojinbaka’, accumulates lots of sodium in leaves. It forms sodium-binding starch granules in the chloroplasts that trap and detoxify sodium in the cytosol of mesophyll cells (Noda *et al.* 2023). Besides “Tojinbaka”, there might be other accessions harboring different mechanisms of salt tolerance. Previously, 28 accessions of *V. riukiuensis* have been assessed for salt tolerance by wilting rate (Yoshida *et al.* 2016). However, most of the accessions could not survive after two weeks (Yoshida *et al.* 2016). Moreover, NARO Genebank has introduced more collections of *V. riukiuensis* (Muto *et al.* 2015, Takahashi *et al.* 2014, Tomooka *et al.* 2013), which have not been evaluated yet.

Therefore, in this study, to explore the potential of salt tolerance in *V. riukiuensis*, we assessed salt tolerance of 61 accessions in a condition of 200 mM of NaCl for a duration of four weeks. Our study showed 16 accessions maintained their effective quantum yield until the end of evaluation. The following analyses revealed further variations in the pattern of Na^+^ allocation even within the most tolerant group. This study indicates that there are other mechanisms of salt tolerance than the sodium-trapping starch granules.

## Materials and Methods

### Plant materials and growth condition

A total of 61 single seed descendant lines of *V. riukiuensis* (Supplemental Table 1) were obtained from the Research Center of Genetic Resources, National Agriculture and Food Research Organization (NARO), Japan (NARO Genebank: https://www.gene.affrc.go.jp/index_en.php). Of these 61 accessions, one is collected from Taiwan and the others are from Okinawa islands of Japan. 53 out of 61 accessions have GPS information (Fig. 1).

Seeds were sterilized with 70% ethanol and 5% sodium hypochlorite for five minutes each and then rinsed thoroughly with tap water. Sterilized seeds were scratched and germinated on Seramis granules (Effem GmbH, Verden, Germany). Germinated seedlings were transferred to four-liter containers containing culture of 1× Otsuka house No. 1 and 1× Otsuka house No. 2 (Otsuka Chemical Co., Osaka, Japan: N, P, K, Ca, and Mg = 18.6, 5.1, 8.6, 8.2 and 3.0 mEq L^–1^, respectively). Each container held six seedlings. 61 accessions were transferred to 31 containers with three biological replications. Plants were grown in a walk-in growth chamber at 28 ^o^C with condition of 14 h light and 10 h dark. After two weeks, 200 mM of NaCl was treated with a renewed hydroponic culture as described above. For the subset of 11 accessions, 3–9 individual plants were used for a second evaluation and for measuring Na^+^ and K^+^ concentrations.

### Chlorophyll fluorescence measurement

Chlorophyll fluorescence was measured using a portable JUNIOR-PAM fluorometer (Heinz Walz GmbH, Pfullingen, Germany) and Photosystem II (PSII) effective quantum yield (Y(II)) was calculated using WinControl-3 software (Heinz Walz GmbH). The measurement was carried out using a leaf clip with a measuring light generated by blue actinic illumination at 190 μmol m^–2^ s^–1^. For each accession, at least one leaflet of the second leaf from the bottom was measured on three individual plants at 0, 1, 3, 7, 14, 21 and 28 days after treatment with 200 mM NaCl. Clustering was performed on the Y(II) values using Euclidean distance and hierarchical agglomeration. For the second experiment on a subset of accessions, the measurement extended to 35 days after salt treatment.

### Ion assessment

For the subset of 11 accessions, leaves, stems and roots were separately collected for ion assessment. Collected samples were dried at 50°C for one week and weighed. Samples were digested with 69% HNO_3_ for the determination of sodium (Na^+^) and potassium (K^+^), by inductively coupled plasma-mass spectrometry (ICP-MS, NexION 350S, PerkinElmer, Waltham, MA, USA).

### Lignin staining and Casparian strips observation

Accessions JP254554 classified as tolerant and JP254537 as sensitive were studied for Casparian strips formation. Growth condition and salt treatment were the same as mentioned above. Roots were sampled 7 days after treatment of 200 mM with NaCl. Collected root samples were kept in 70% ethanol at 4℃ until sectioning. The primary roots were sliced using a sharp double-edged razor blade under a stereomicroscope (Leica MZ16, Leica Microsystems) at 1, 3, 5, 7 and 9 cm from the root apex. Lignin was stained by a mix of 3% phloroglucinol and 35% hydrochloric acid in a volume ratio of 2:1. The stained root sections were immediately observed using bright-field microscopy (Axioskop 2 plus, Zeiss).

## Results

### Variation of chlorophyll fluorescence in *V. riukiuensis* at 200 mM of NaCl

To investigate intraspecific variation, we evaluated the chlorophyll fluorescence of 61 *V. riukiuensis* accessions as an indicator of salt tolerance. Based on the effective quantum yield (Y(II)) under salt stress, accessions were classified into five clusters (Fig. 2a, cluster 1 to 5), representing a gradient from high to low salt tolerance (Supplemental Fig. 1). Accessions in cluster 1 maintained Y(II) for four weeks in 200 mM of NaCl, indicating their photosynthetic activity was not affected by salt stress (Govindjee 1995), whereas those in cluster 5 showed the earliest decline. Accessions in cluster 2, 3, 4 maintained Y(II) for three, two and one weeks, respectively (Fig. 2a). No correlation was found between the island habitat and salt tolerance (Supplemental Table 1).

To assess the reproducibility of the above results, we selected a subset of accessions from each cluster and assessed Y(II). Specifically, we randomly selected three accessions from cluster 1 and two accessions each from the other clusters. Overall, the results were consistent with the initial screening in terms of the classification of salt tolerance among accessions, with two accessions showing minor variation (Fig. 2b). While JP254490 maintained Y(II) for four weeks in the initial screening but only for three weeks in the second experiment, JP254504 showed the opposite trend—extending the maintain of Y(II) from two weeks initially to four weeks in the second experiment.

### Na^+^ and K^+^ accumulation across tissues

To investigate tissue accumulation patterns and correlations between ion concentrations and salt tolerance, the above subset of accessions was subjected to assessment of sodium (Na^+^) and potassium (K^+^) concentrations in roots, stems, and leaves.

For most accessions, the three organs of each accession showed no difference in Na^+^ accumulation (Fig. 3a), except JP254554 in cluster 1 and JP254537 in cluster 5. The former had significantly higher Na^+^ in roots than in leaves whereas the latter accumulated higher Na^+^ in stems and leaves than in roots (Fig. 3a).

Regarding K^+^ concentrations, each accession accumulated less in stems than in roots and leaves, although the difference from leaves was not significant for all accessions (Fig. 3b). K^+^ concentrations were significantly higher in leaves than in roots in accessions JP254554 (cluster 1), JP254529 (cluster 2), JP254530 (cluster 3), JP254487 (cluster 4), JP254525 and JP254537 (cluster 5) (Fig. 3b).

By using the Y(II) values assessed for the subset of accessions in the second experiment, we calculated the area under the curve (AUC) of Y(II) over time as an indicator of salt tolerance, where higher values reflect better maintenance of photosynthetic efficiency under salt stress. And we studied whether salt tolerance is associated with ion concentration. As a result, leaf Na^+^ concentrations were not correlated with salt tolerance (Table 1). In contrast, the leaf K^+^ and root K^+^ concentrations were positively correlated with AUC (Table 1), indicating that salt-tolerant accessions have higher ability of K^+^ retention in these organs. A moderate positive correlation between AUC and root Na^+^ concentration (r = 0.57, *p* = 0.067) was also observed, although it was not statistically significant at the 0.05 level (Table 1).

### Observations on root apoplastic barriers

We noticed that the tolerant accession JP254554 and the sensitive accession JP254537 exhibited contrasting patterns of Na^+^ distribution. JP254554 retained Na^+^ in the root and suppressed the Na^+^ accumulation in the leaf, whereas JP254537 did not hold Na^+^ in the root and accumulated high levels of Na^+^ in the leaf (Fig. 3a). Since our previous study revealed the importance of root apoplastic barrier in excluding Na^+^ from aerial tissues in *Vigna marina* (Wang *et al.* 2025), we were prompted to ask whether JP254554 employs a similar mechanism. We prepared root sections of the two accessions, stained lignin and observed by microscopy. Not as we expected, JP254554 developed much less root barrier in the endodermis compared to the sensitive accession JP254537 (Fig. 4). No observable root barriers were formed up to 3 cm from the root tip and only weak signatures of Casparian bands in 5–9 cm from the root tip. In contrast, JP254537 showed stronger staining in any sections we observed, especially at the position of 3 cm from the root tip (Fig. 4).

## Discussion

This study revealed substantial intraspecific variation of salt tolerance in *V. riukiuensis*. Previous studies have reported *V. riukiuensis* as a salt-tolerant species based on a single accession (Iseki *et al.* 2016, Noda *et al.* 2022, 2023). However, in this study, JP254537, which is a single-seed descendant of the accession tested in the former studies, was revealed to be one of the least-tolerant accessions (cluster 5) (Fig. 2, Supplemental Fig. 1). While the cluster 5 accessions have lost chlorophyl fluorescence within a week, those with the highest tolerance (cluster 1) maintained it for more than 4 weeks. Thus, we have identified potentially better donors for breeding salt-tolerant adzuki bean. Moreover, we found the accessions in cluster 1 have acquired the supreme salt tolerance by currently unknown mechanisms. For example, JP254554 is able to suppress Na^+^ transport to the leaves without developing Casparian strips (Fig. 4).

Our results suggested salt tolerance may have evolved independently in the isolated populations. Iseki *et al.* (2016) argued that salt tolerance could easily occur at species level in the genus *Vigna*, as salt-tolerant species did not form a single clade but were distributed across multiple clades in the phylogenetic tree. However, in this study, the degree of salt tolerance greatly varied even among the accessions collected from the same island (Supplemental Table 1). Given our recent studies revealed that altering a few genes have substantially contributed the evolution of salt tolerance in wild species (Ito *et al.* 2024, Noda *et al.* 2025, Wang *et al.* 2025), we consider it could also happen in some isolated populations of *V. riukiuensis*.

Although the variation in leaf Na^+^ allocation is often associated with intraspecific variation in salt tolerance (Munns and Tester 2008, Schachtman and Munns 1992), we did not see a clear correlation among the accessions we tested. As in Fig. 3a, both the clusters 1 and 5 contained accessions with high and low Na^+^ allocation to the leaves. This suggests that Na^+^ concentration alone does not explain salt damage; instead, Na^+^ sequestration in vacuoles or other organelles may also play some roles in salt tolerance. Notably, the Na^+^ concentrations that accessions could tolerate varied substantially, as even relatively low Na^+^ levels resulted in salt sensitivity in JP254525 and JP254487 (Fig. 3a). Meanwhile, K^+^ contents in roots and leaves showed a positive correlation with salt tolerance (Table 1). As K^+^ is one of the essential nutrients for a plant, the ability of K^+^ uptake under salt stress could contribute to the maintenance of chlorophyll fluorescence. However, the correlation was weak and thus it cannot solely explain the whole variations in salt tolerance across the accessions.

The results of root barriers were also an unexpected one. As JP254554 in the cluster 1 and JP254537 in cluster 5 showed a contrasting pattern of Na^+^ allocation, we expected JP254554, which has restricted Na^+^ allocation to the leaf, has thicker root barriers than JP254537, which has allocated Na^+^ in the leaf but not in the root (Fig 3a). However, the result was totally opposite: JP254554 hardly formed root barriers while JP254537 had much thicker one. We were surprised by the result, as we had shown that the root barriers were indispensable in sodium-excluding ability of *V. marina*, the most salt-tolerant species in the genus (Wang *et al.* 2025). Thus, our results suggest that lignin deposition in endodermis needs another factor to prevent apoplastic transport of Na^+^. What was more surprising was the JP254554 achieved the low Na^+^ allocation to the leaf without the root barrier. We do not currently know its mechanism, but our future studies using the mapping population derived from JP254537 × JP254554 will elucidate the query.

To summarize, we have revealed great diversity of salt tolerance in *V. riukiuensis*. We have identified accessions that are even more tolerant than the one selected in former studies. In addition, many of them have acquired the higher salt tolerance by currently unknown mechanisms. We hope this study draws interests of not only breeders but also scientists who are willing to elucidate how such diverse mechanisms of salt tolerance have evolved.

## Author Contribution Statement

FW and KN conceived the study and wrote the paper. FW and YI measured chlorophyll fluorescence. KT measured ion contents. FW observed root sections.

## Supplementary Material

Supplemental Figure

Supplemental Table

## Figures and Tables

**Fig. 1.**
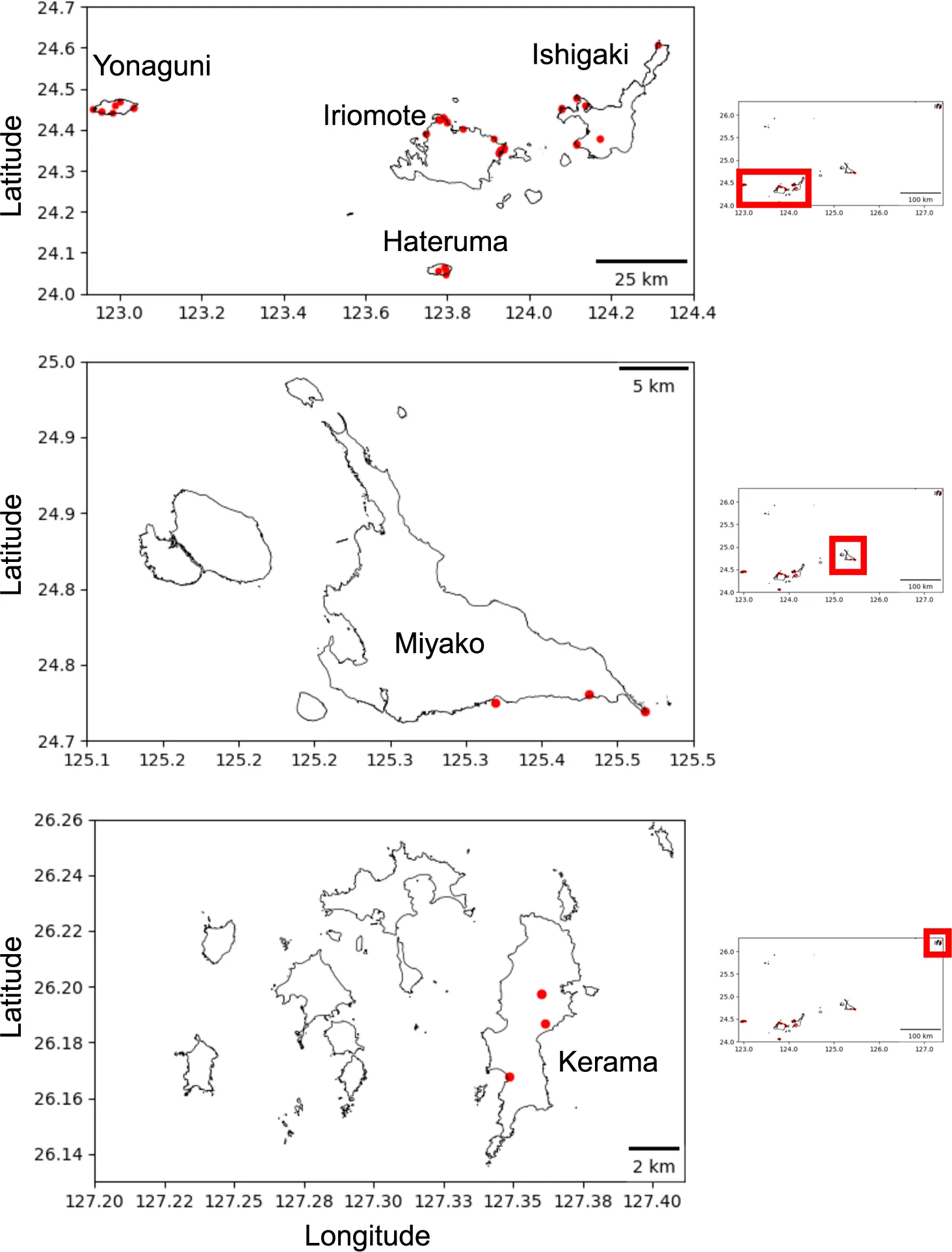
Collection sites of 53 accessions of *V. riukiuensis*.

**Fig. 2.**
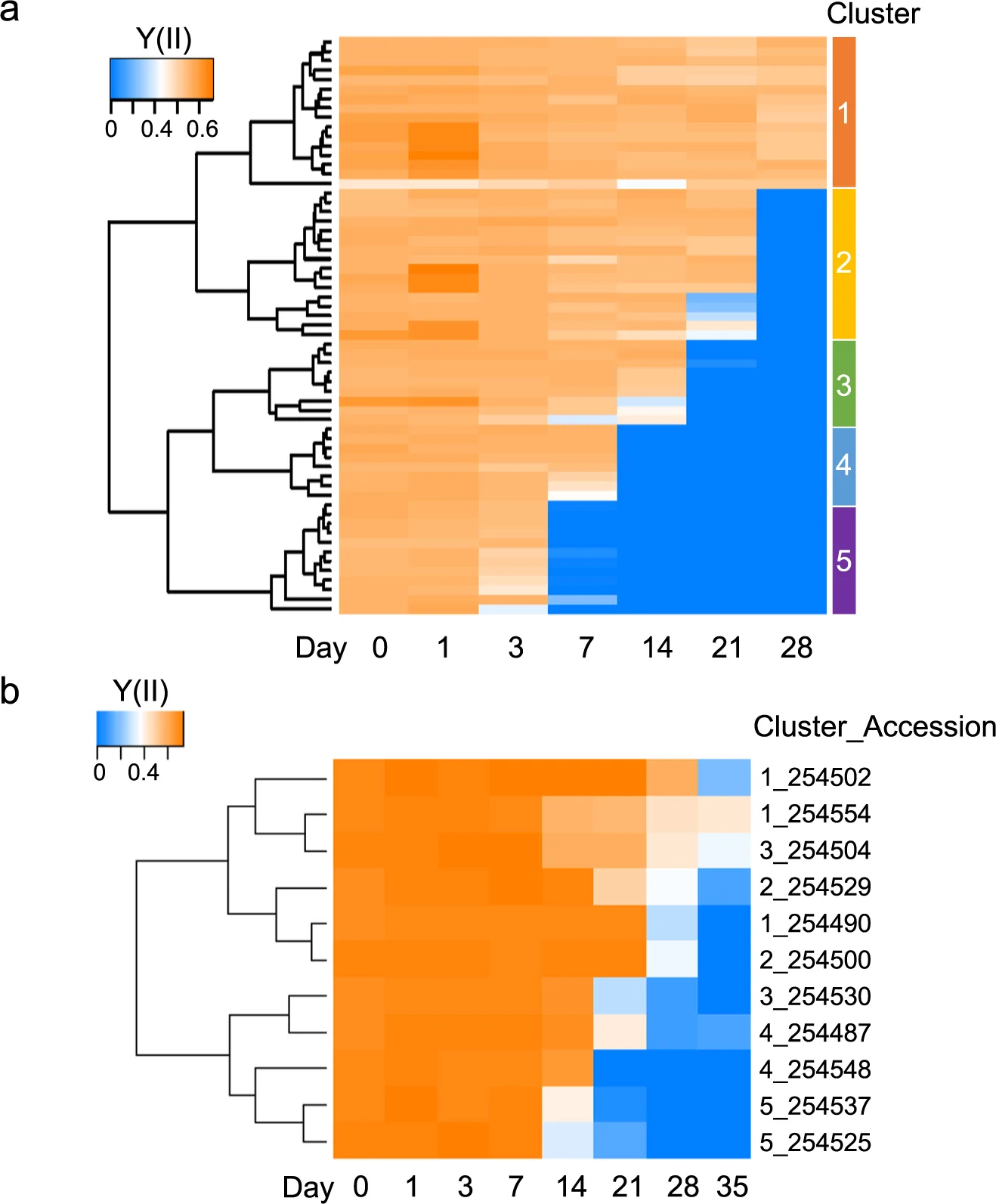
Time course change of effective quantum yield (Y(II)) under 200 mM NaCl of a) 61 accessions and b) a subset of accessions of *V. riukiuensis*. The number before the underscore indicates the cluster number, and the number after the underscore represents the JP accession number in b). Clustering was performed on the Y(II) values using Euclidean distance and hierarchical agglomeration.

**Fig. 3.**
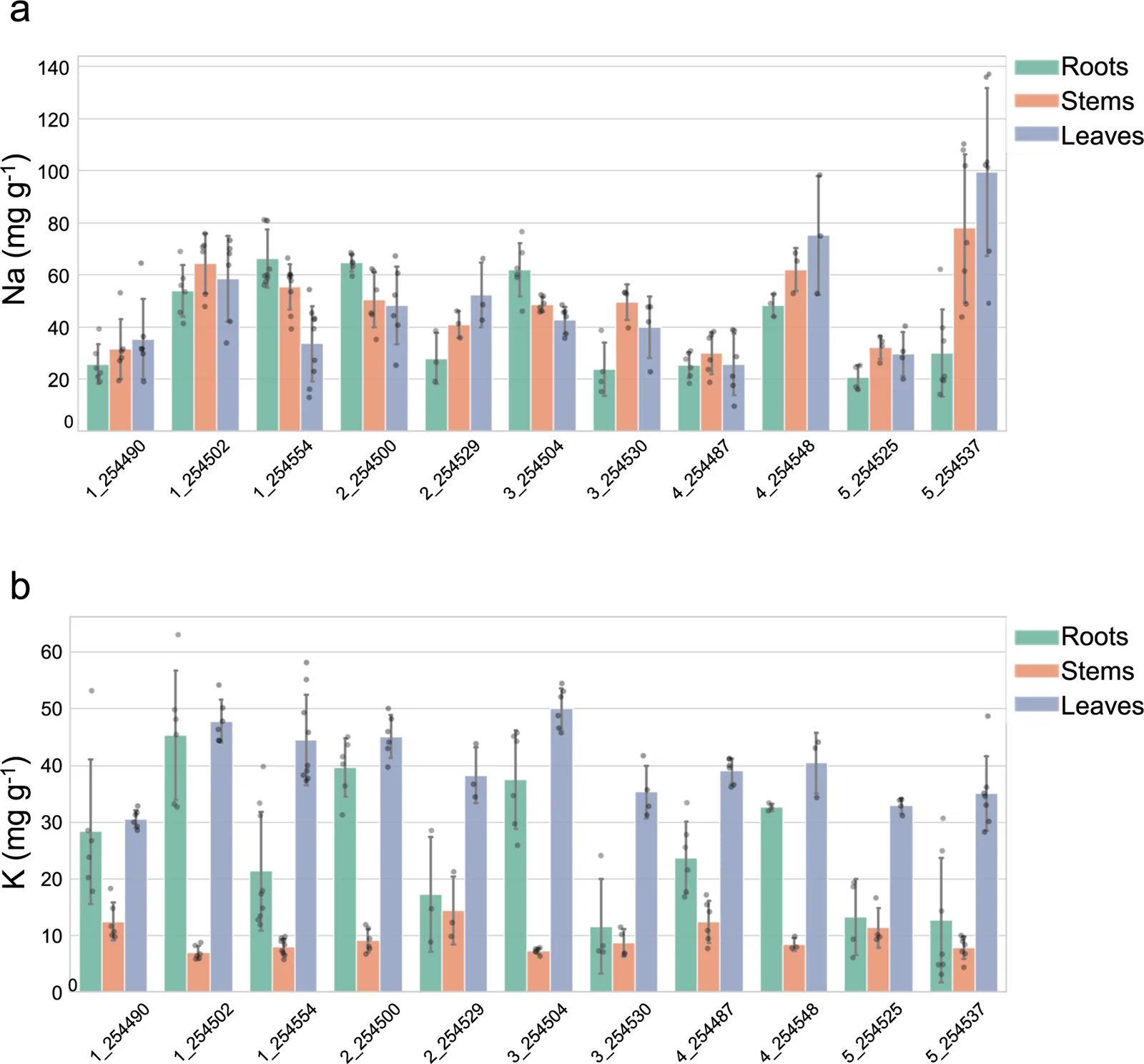
Sodium (Na^+^) (a) and Potassium (K^+^) (b) concentrations in roots, stems and leaves of a subset of *V. riukiuensis* accessions. Dots indicate data collected from 3–9 individual plants and error bars indicate standard deviation. On x-axis, the number before the underscore indicates the cluster number, and the number after the underscore represents the JP accession number.

**Fig. 4.**
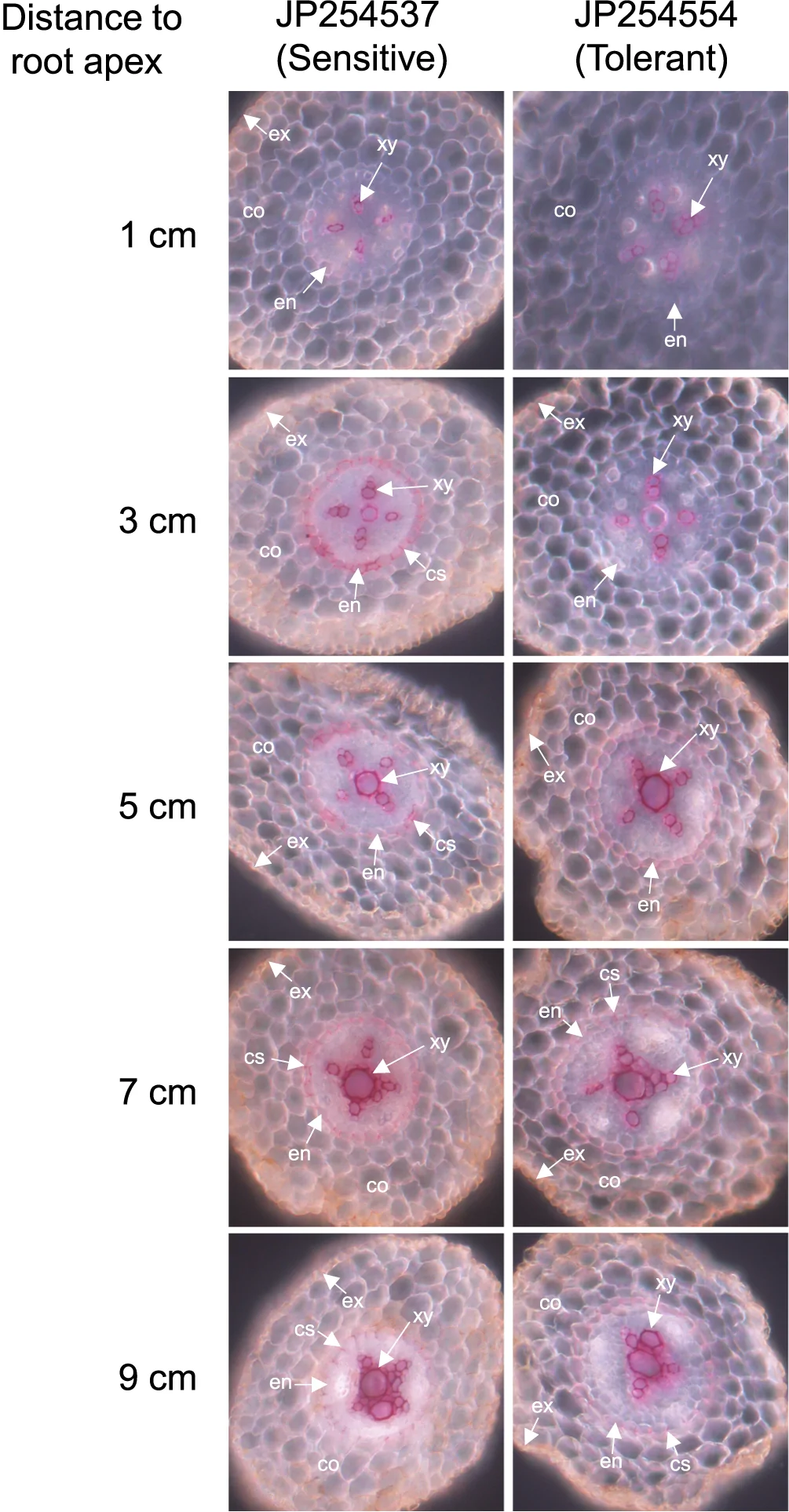
Lignin staining for Casparian strips observation of JP254537 and JP254554 at 1, 3, 5, 7 and 9 cm from the root apex at 7 days after treatment of 200 mM of NaCl. ex: exodermis, co: cortex, en: endodermis, cs: Casparian strips, xy: xylem. Casparian strips at endodermis and xylem vessels are stained.

**Table 1.** Correlation between AUC of Y(II) and ion concentrations

Ion	Tissues	Correlation (r)	*p*-value
Na^+^	Root	0.571	0.0667
	Stem	–0.11	0.7483
	Leaf	–0.35	0.291
K^+^	Root	0.651	0.0299*
	Stem	–0.075	0.8271
	Leaf	0.626	0.0392*
Na^+^/K^+^	Root	–0.124	0.7167
	Stem	0.004	0.9907
	Leaf	–0.516	0.1045

* Significant at *p* < 0.05.
