# Supplementary material for: Diversity of salt tolerance in Vigna riukiuensis
Source: Breed Sci. 2026 Jun 11;76(3):318–24. doi: 10.1270/jsbbs.25064 (PMC13709618; doi:10.1270/jsbbs.25064)
Supplement: Supplementary file 1 [file 76_318_s1.pdf]

Wang et al./Breeding Science
doi: 10.1270/jsbbs.25064

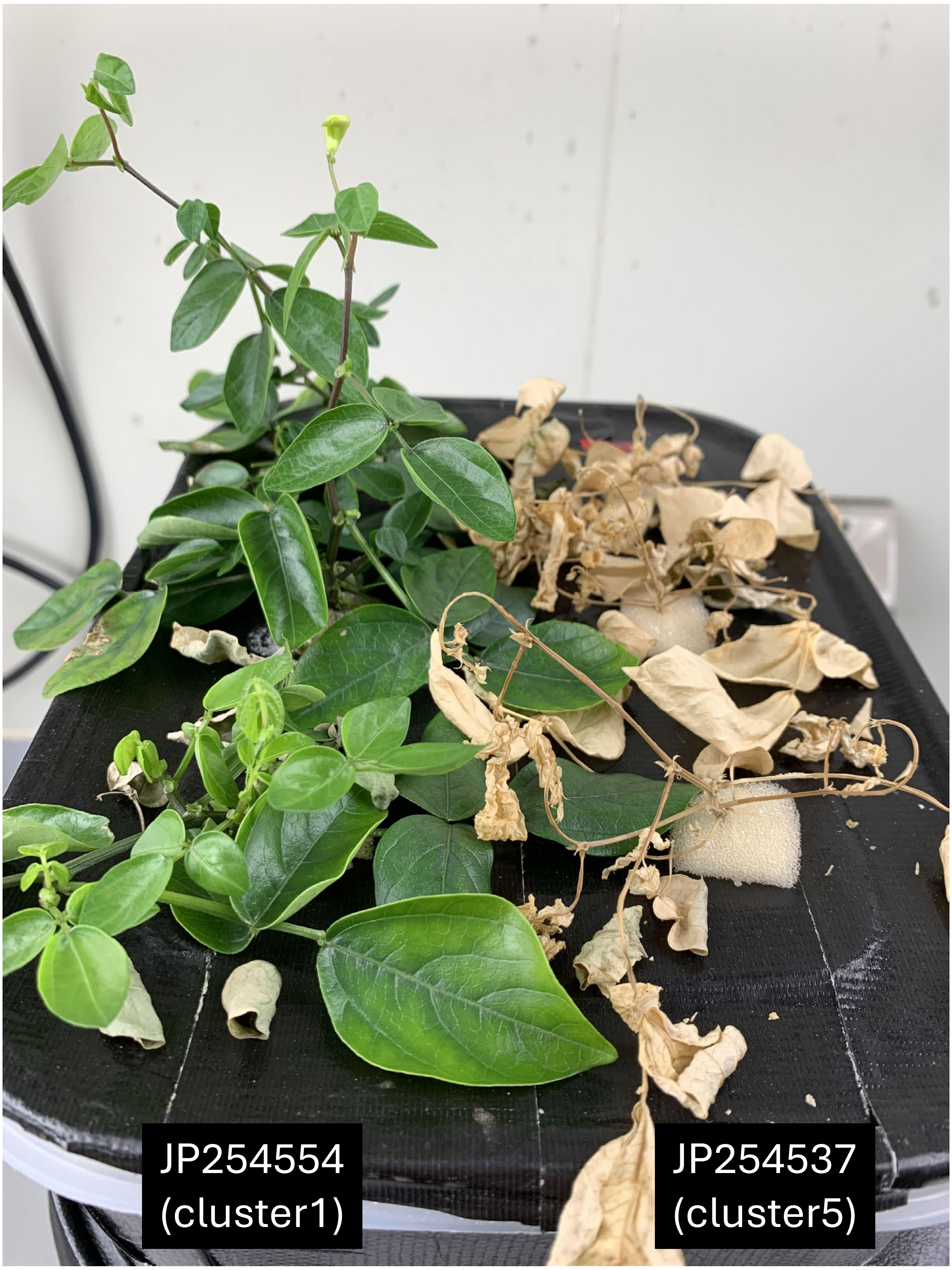

Supplemental Fig 1. Photo of the salt-tolerant and salt-sensitive accessions. The photo was taken on the day 21 after initiation of salt stress.
